# The Generation of Wind Velocity via Scale Invariant Gibbs Free Energy: Turbulence Drives the General Circulation

**DOI:** 10.3390/e27070740

**Published:** 2025-07-10

**Authors:** Adrian F. Tuck

**Affiliations:** Independent Researcher, Boulder, CO 80303, USA; adrianftuck@gmail.com

**Keywords:** airborne observations, statistical multifractals, molecular collisions, wind velocities

## Abstract

The mechanism for the upscale deposition of energy into the atmosphere from molecules and photons up to organized wind systems is examined. This analysis rests on the statistical multifractal analysis of airborne observations. The results show that the persistence of molecular velocity after collision in breaking the continuous translational symmetry of an equilibrated gas is causative. The symmetry breaking may be caused by excited photofragments with the associated persistence of molecular velocity after collision, interaction with condensed phase surfaces (solid or liquid), or, in a scaling environment, an adjacent scale having a different velocity and temperature. The relationship of these factors for the solution to the Navier–Stokes equation in an atmospheric context is considered. The scale invariant version of Gibbs free energy, carried by the most energetic molecules, enables the acceleration of organized flow (winds) from the smallest planetary scales by virtue of the nonlinearity of the mechanism, subject to dissipation by the more numerous average molecules maintaining an operational temperature via infrared radiation to the cold sink of space. The fastest moving molecules also affect the transfer of infrared radiation because their higher kinetic energy and the associated more-energetic collisions contribute more to the far wings of the spectral lines, where the collisional displacement from the central energy level gap is greatest and the lines are less self-absorbed. The relationship of events at these scales to macroscopic variables such as the thermal wind equation and its components will be considered in the Discussion section. An attempt is made to synthesize the mechanisms by which winds are generated and sustained, on all scales, by appealing to published works since 2003. This synthesis produces a view of the general circulation that includes thermodynamics and the defining role of turbulence in driving it.

## 1. Introduction

Hydrodynamic flow was shown to emerge spontaneously, by computer simulation, from a thermalized population of hard spheres—billiards—subject to an anisotropic, symmetry breaking energetic flux of such particles [1,2]. The continuous translational symmetry of a Maxwell–Boltzmann population had been broken. The emergent flow will be referred to as organized rather than ordered, in accord with the remarks of references [3,4], although the term ‘order’ appears to have originated with Gibbs. The word ‘heat’ will be treated as a verb, not as a noun, per pages 22–28 in [3]. ‘Thermal energy’ will be used. The idea that the highest energy molecules carry Gibbs free energy and the associated ability to conduct entropy-free work is taken from [5,6]. The emergence and maintenance of jet streams has uncertainties [7], as do the very high wind speeds in tropical cyclones (hurricanes, typhoons) and tornadoes. The application of the techniques of statistical multifractality [8] to airborne and dropsonde observations [9,10] led to an unexpected correlation between the intermittency of temperature and both the ozone photodissociation *rate* and temperature itself [11,12]. The correlation was with the product of the photodissociation coefficient and the ozone number density; there was none with the individual *J* and [O_3_] factors. Note that the acceleration of air molecules from an energy source can occur from the extreme of the shock wave formation produced by lightning to more routine sources such as sea surfaces and high-relative-humidity land terrains in the troposphere, in addition to the energy from photofragments. In all cases the high velocity molecules lead, with lower-velocity molecules following. The average molecules act to eliminate the number density gradient, and in doing so, set up a vorticity pattern, a ‘ring current’. In the atmosphere, unlike in a laboratory apparatus, the high-velocity molecules will encounter vortical distributions at whatever scale is being considered, so the high velocities tend to bunch together and contribute to an acceleration which is limited by the ability of the average molecules to create entropy by emitting IR radiation into space. In practice these higher velocities will be less than the speed of sound of thunder from a lightning shock wave but can reach 100 ms^−1^ in upper tropospheric jet streams and more in the stratosphere [11]. Very high velocities are thought to occur in some tropical cyclones and in some tornadoes. We review the evidence for the proposed mechanism producing accelerations to such high velocities. References [9,10,11,12,13,14,15,16,17,18,19,20] are involved, with [6,9,11], providing more detailed arguments. References [21,22] are relevant to arguments about the role of molecular behavior in connecting the microscopic and macroscopic scales—the “middle way”.

*“Does the wind possess a velocity? This question, at first sight foolish, improves on acquaintance”.*

L. F. Richardson [23].

## 2. Materials and Methods

The observational data used here were taken by the NASA ER-2, DC-8, and WB57F aircraft and by the NOAA Gulfstream 4SP equipped with GPS dropsondes. More details can be found in references [11,13]. When the DC-8 is included, the coverage is pole to pole. The ER-2 was flown between 72° S and 84° N, while the WB57F flew between 46° N and the equator. The Gulfstream 4SP flew between 15° N and 60° N over the eastern Pacific Ocean.

The statistical multifractal analysis has been described in [8,11]. The mappings between equilibrium statistical mechanics variables and their scaling equivalents are listed in Table 1 below; the derivations may be seen in [8,14].

The variables are obtained as follows. *q* defines the *q*th order structure function of the observed quantity. The scaling exponent *K*(*q*) is derived from the slope of a log–log plot [8,11,14].

(1) H=H(q)+K(q)/q

and an analysis of energy *E* in terms of a scale ratio produces the fractal co-dimension *c*(*γ*). *C*_1_ is the co-dimension of the mean, specifying the generator of the intermittency that is the logarithm of the turbulent flux. For a real system such as the atmosphere, its value may not be confined to the theoretical range. The means converge but the variance does not, a result expressed as 2.0 > α >1.5. Intermittency as computed applies over all observed scales, 40 m to an Earth radius. It is consistent with the concentration of thermal energy into local vorticity structures. *H* is the conservation exponent, sometimes called the Hurst exponent. It is derived and described in [6,8,11,14]. *H* = 0 is uncorrelated on all scales, 0.5 is random, and 1 is perfect correlation. Intermittency, as seen here, complicates the interpretation.

The dropsonde data were analyzed for temperature, relative humidity, and wind velocity [15].

## 3. Results

**Figure 1:**
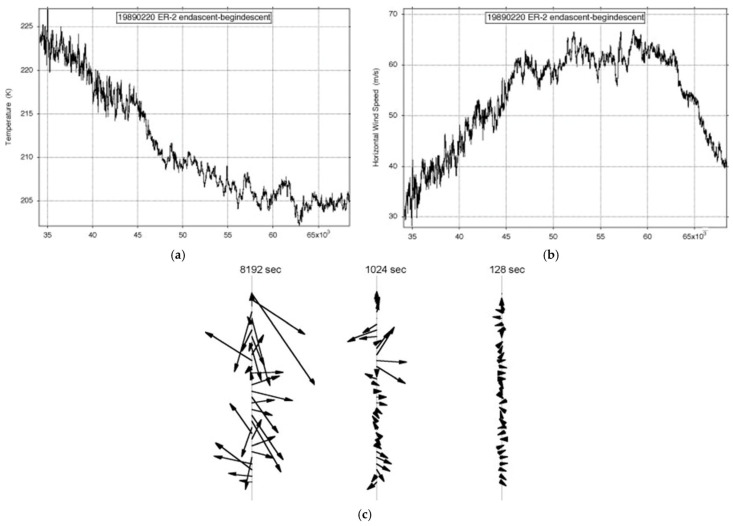
ER-2 flight from (59° N, 6° E) to (38° N, 75° W) on 19890220: (**a**) temperature, (**b**) wind speed, (**c**) shear vectors averaged over intervals differing by a factor of 2^3^. The length of the flight exceeded an Earth radius; the airplane flew at 200 ms^−1^. The flight path was along the jet stream, a head wind along a distance slightly longer than an earth radius. The shear vectors occurred at all angles, and the variance of the wind velocities did not converge. This appears as Figure 7 in [11]. A higher-definition version is constituted by Figures 2.1, 2.2, and 2.3 in [6].

**Figure 2:**
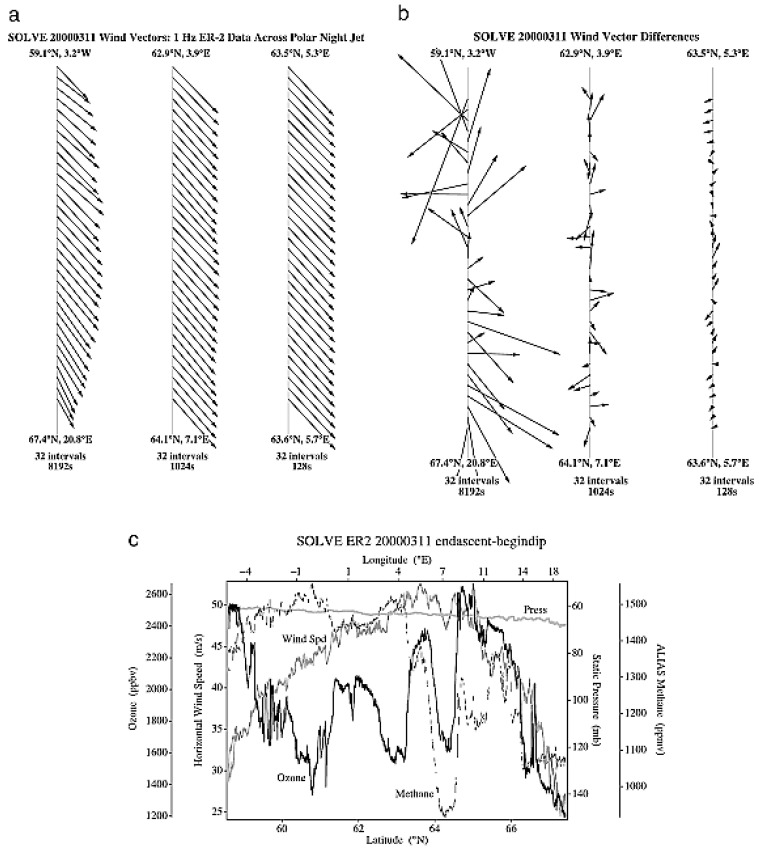
The arrows in (**a**) represent the vector winds experienced by the ER-2 in crossing the polar night jet stream between (71° N, 20° E) and (59° N, 4° W) on 20000311. They are given over three intervals of 8192, 1024, and 128 s, each centered on the jet axis. The corresponding vector differences are shown in (**b**) and demonstrate the inevitability of positional exchange between neighboring intervals on all scales. Note the structure in methane and ozone across the jet (**c**). It appears as Figure 1 in [16].

The basic structure of jet streams as observed by the ER-2 is exemplified in Figure 1 and Figure 2. Examples are shown from both AASE (1989) and SOLVE (2000), respectively. The 1989 flight is along the jet stream, and the 2000 flight is across it. There is scale invariance in the wind, and the associated shear vectors rotate through 360° in the plane of the aircraft flight track. The concurrent temperature field shows similar variability in these long flight legs. The observational data are averaged to 1 Hz, corresponding to approximately 200 m; the meteorological data can support averaging to 5 Hz, or 40 m with similar statistical characteristics. More detailed descriptions can be found in references [6,11]. The shear vectors in Figure 1 and Figure 2 were calculated as the resultant of the first differences of the north–south and east–west wind speeds [15]. As with all airborne in situ observations, the results are calculated along the flight track. Scale invariance is evident when arbitrary selection of a ‘window’ on the abscissa in the absence of its numerical intervals leaves no idea of the scale selected.

**Figure 3:**
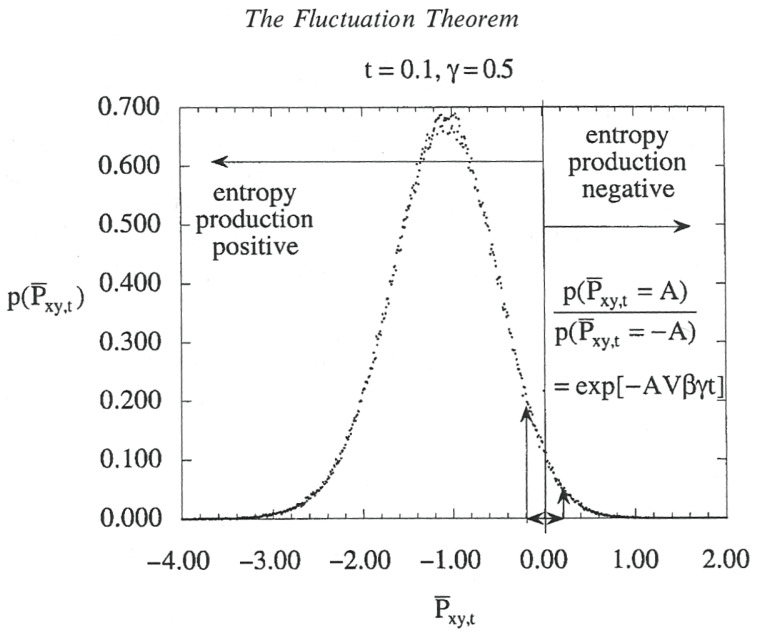
How the faster molecules produce ‘organization’, negative entropy production, while the slower, more probable ones produce positive entropy. This is from [5] and is the expectation from sheared flow in a molecular gas under a constant strain rate in the *x*-direction *γ* = ∂*u_x_/∂y*; the system is at a constant volume and at a constant temperature, *T*. The temporal averages of the *xy*-element of the pressure tensor, <*P_xy,t_*>, are proportional to minus the time-average of the entropy production, and label the abscissa. The dots comprise the histogram of the probabilities for <*P_xy,t_>*; the ratio of the positive to negative probabilities, *p*, declines exponentially with volume, strain rate, and time; negative entropy production is local and short-lived. Thus, although the production of ‘organization’ in fluctuations with negative entropy production is small, it is not zero. Note that the emergence of vortices in [2] is an example of fluid flow (‘organized’) emerging as the result of mutually sustaining feedback between the faster molecules and the ‘ring currents’. The real atmosphere is not under a constant strain rate, is not at a constant temperature, and experiences anisotropies from gravity, planetary rotation, the solar beam, and the planetary surface. Fluid flow in the atmosphere emerges via the ‘ring current’ mechanism as translationally hot ozone photofragments recoil not into a thermalized bath but into a pre-existing set of scale invariant vorticity structures from 10^−9^ to 10^7^ m. In the real atmosphere the histogram would therefore be expected to have a non-Gaussian shape, with a power law tail rather than an exponential one at negative entropy productions. The peak values still correspond to dissipation (positive entropy production) and permit the operational definition of atmospheric temperature. See [5,6] for further details. This appears with permission as Figure 7.1 in [6].

The thermodynamic behavior of a population of hard spheres, in a numerical simulation, subject to a constant strain in the *x*-direction at a constant volume is shown in Figure 3. The production of organized flow, ‘negative entropy’, is carried by the most energetic (fastest) molecules while the more numerous average molecules produce ‘positive entropy’ and in so doing define an operational temperature [5,6]. The organized flow is not long-lived in this laboratory-like simulation, but it illustrates the principle that the translationally ‘hot’ molecules propagate the flow. In the real air as sampled by the aircraft, an analysis at constant pressure involves Gibbs free energy [14] and fat tails in the PDF (probability distribution function). The majority of the molecules exchange energy easily, and in doing so define an operational temperature. Although the most energetic molecules are a minority, they nevertheless shape the flow and perform entropy-free work that sustains the flow.

**Figure 4:**
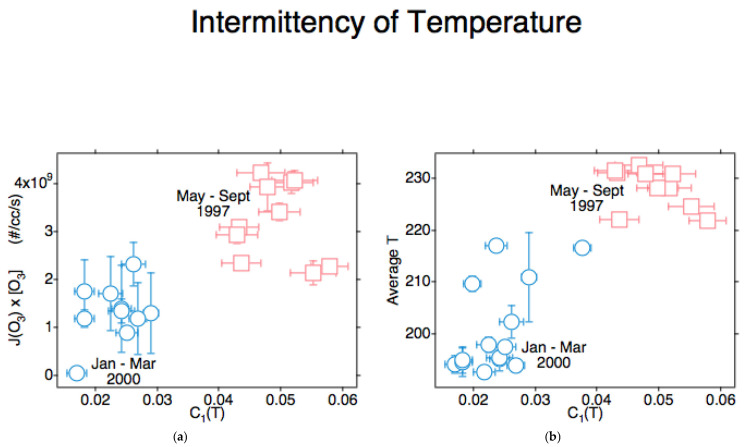
The correlation of the intermittency of air temperature with (**a**) ozone photodissociation *rate* and with (**b**) temperature itself. There is no correlation with *J* alone or with [O_3_] alone. The observations were taken from the ER-2 at about 55 mbar during Arctic summer 1997 and Arctic winter 2000. This appears as Figure 16 in [11].

The observational result leading to the conclusion that air molecules were not at thermal equilibrium was the intermittency of temperature correlated with the ozone photodissociation *rate*, and with temperature itself [6,9,12], as shown in Figure 4. The analysis was performed by statistical multifractal techniques, with the scaling variables as defined in Table 1. The flights were by the ER-2 in the Arctic lower stratosphere in summer 1997 and winter 2000 in the lower stratosphere. The scatter in the points is consistent with the non-convergence of the variance and with changes in the air masses over the summer and winter periods. Nevertheless, the correlation is clear and makes physical sense. An explanation was sought for this unexpected result and found in [2]: the emergence and propagation of hydrodynamic flow in a population of hard spheres whose continuous translational symmetry was broken by a flux of such energetic particles. An equilibrated population has continuous translational symmetry.

**Figure 5:**
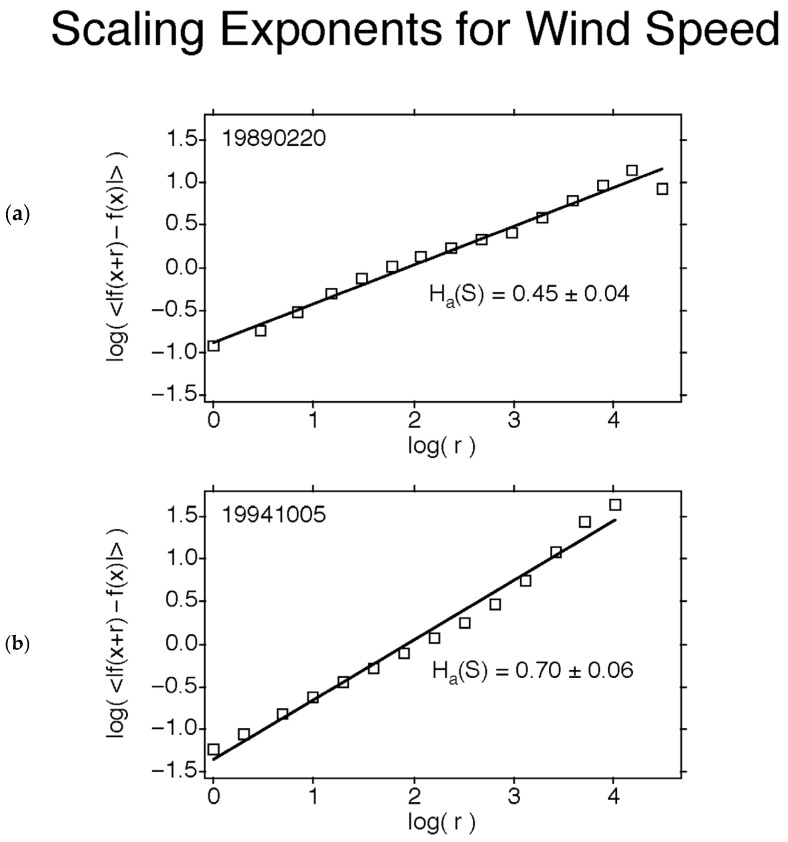
The scaling exponent *H* for wind speed for extremal values in the stratospheric polar night jet in (**a**) Arctic and (**b**) in the Antarctic. Observations taken from the ER-2. (**a**) is along the jet while (**b**) is across the jet. See Figure 1 as regards (**a**), and [24] as regards (**b**).

For operational reasons, most ER-2 flights were poleward across the lower stratospheric polar night jet stream instead of being along it. Figure 5 shows examples of the scaling of wind speed (a) along the jet into a headwind and (b) across the jet. There is more organization across the jet along it, interpreted as meaning that speed shear is more effective at producing variation—turbulence—than is directional shear [11,15,16]. Dissipation is by infrared emission from the entire atmosphere comprising the open thermodynamic system, at operationally defined temperatures. It is not meaningful to speak of dissipation by turbulence in a locally defined context. Further discussion occurs in Section 4.3 below. The wind speeds and altitudes were similar in the two flight legs. This appears as Figure 8 in [11].

The relationship of statistical multifractal scaling analysis exponent *H* to spectral analysis quantity *β* is discussed in [25]. *β* = 2*H* + 1 if intermittency can be ignored, which for the observations discussed here, it cannot. See also [17]. Spectral analysis imposes a symmetry on air that it does not have.

**Figure 6:**
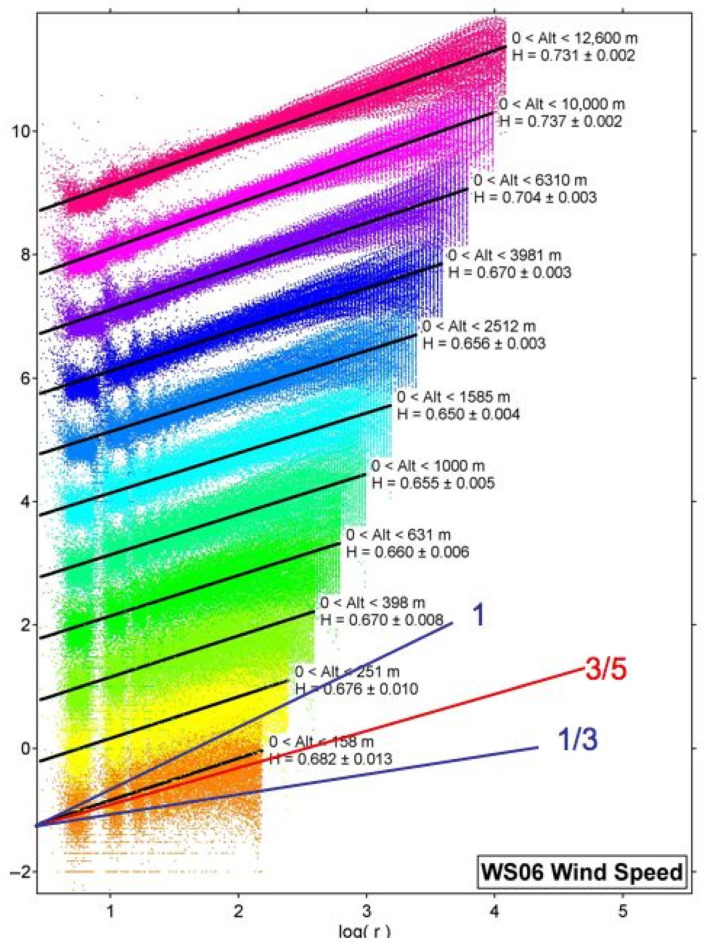
The vertical variation of the exponent *H* of the wind speed in the horizontal plane observed by 315 GPS dropsondes from the Gulfstream 4SP during January–March 2006 over the Pacific Ocean area defined by (21°–60° N, 128°–172° W). Each layer of colored points shows *H.* The black lines are the r.m.s. fits to the vertical shear of the respective layers. They increase upwards logarithmically. Note that *H* increases vertically upwards, approaching 0.75 at 12.6 km, where jet streams are prevalent. The lines labeled 1, 3/5, and 1/3 are the theoretical values for gravity waves, Bolgiano–Obukhov, and Kolmogorov, respectively. Isotropy, Kolmogorov, was never observed. See [10,18] for further explanation. This appears as Figure 3 in [11]. A higher-definition black-and-white version appears as Figure 4.12 in [6].

The initial reaction to the scaling analysis of the vertical data from the GPS dropsondes during the Winter Storms projects was surprising given the vertical variation in *H* for the horizontal wind, as shown in Figure 6. There was a consistent behavior during all three Winter Storms missions in January–March of 2004, 2005, and 2006 [17,18]. The interpretation offered subsequently [18] was in terms of symmetry breaking of continuous translational symmetry by the persistence of molecular velocity after collision [19]. Reference [18] relates the molecular view to traditional meteorological methods using the thermal wind equation: “The westerlies increase with height because it’s colder towards the poles”. Note that the Navier–Stokes equation cannot be solved analytically and does not account for turbulence [20]. Difficulties arise in numerical model simulations because of the twin problems of representing closure and dissipation. These difficulties are avoided if the problem is approached from the bottom–up, i.e., from molecular scales.

**Figure 7:**
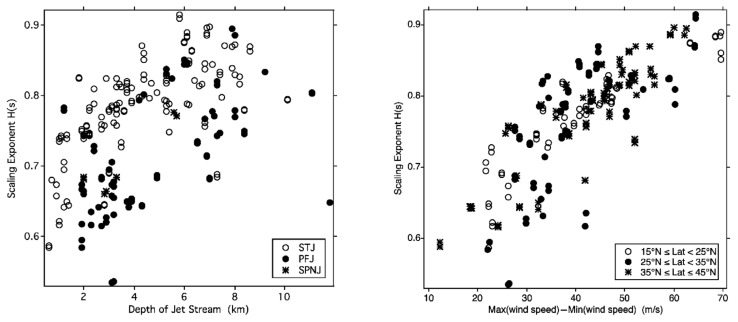
The scaling exponent H in the vertical for the horizontal wind speed calculated from 885 GPS dropsondes from the Gulfstream 4SP during the Winter Storms missions from January to March 2004, 2005, and 2006. (**Left**), versus jet stream depth; (**Right**), versus maximum wind shear. Note the correlation of H with traditional measures of jet stream intensity. This appears as Figure 10 in [11].

The correlation of *H* with conventional jet stream variables can be seen in Figure 7; it applies to all three categories of jet streams encountered by the Gulfstream 4SP during the following missions, the subtropical (STJ), the polar front (PFJ), and the stratospheric polar night (SPNJ). There is more organization (higher *H*) in the subtropical jet stream than in the polar front jet stream, consistent with their synoptic meteorological behaviors. Conventional turbulence theory relies on ad hoc parametrizations rather than analytical solutions of the Navier–Stokes equation and cannot be relied upon to yield a coherent interpretation of the scaling behavior [20]. Note that the energy of the faster molecules carrying the Gibbs free energy is a small fraction of the total but is nevertheless causal in defining wind velocity. That echoes the situation in conventional formulations, where the total kinetic energy is typically in the range of 1–10% of the total energy. See Section 4.3 below for further discussion.

The meteorological observations from the ER-2 are described in [26], which gives details of the calculation of wind speeds and directions relative to the Earth’s geographical Cartesian coordinate system.

The evidence described above, taken from airborne observations combined with a theoretical calculation, points to an absence of thermal equilibrium in air on all scales down to the smallest. Fat-tailed PDFs and statistical multifractal behavior have been revealed, with the fastest moving molecules carrying the scaling version of Gibbs free energy, *G*. What *G* expresses is the ability to conduct entropy-free work and so drives the general circulation of the atmosphere. Although the fastest molecular population is a relatively small fraction of the total, it is the one that is active in producing hydrodynamic flow. It can do so because the remaining larger population defines an operational temperature and pays the entropy cost by infrared radiation into space, which at 2.7 K forms the necessary cold sink.

## 4. Discussion

What is the mechanism by which the translationally hot air molecules carrying Gibbs free energy cause winds up to jet stream velocities? The discussion is divided into four sections, establishing the basic facts and arguments.

### 4.1. Continuous Translational Symmetry and How It Is Broken

A population of Maxwellian ‘billiards’—hard spheres—possesses continuous translational symmetry, because from whatever direction the population is viewed or traversed, or however it is rotated, it looks the same. That property exists because collisions among the members of the population, represented as idealized molecules by Maxwell and Boltzmann, result in recoil with random phase and velocity. The result is thermal equilibrium. In air, the persistence of molecular velocity after collision [19] breaks the continuous translational symmetry. The population of fast-moving molecules is produced by photofragments from ozone photodissociation [12], reinforced by the strong intermittency of the incident solar radiation [27], as illustrated in Figure 4. A local thermodynamic equilibrium does not exist in air. The ultimate symmetry breaker in the planet’s fluid envelope is plate tectonics [22].

### 4.2. Scaling of Temperature and the Middle Way

Temperature should scale like a passive scalar (a tracer) if local thermodynamic equilibrium exists in air. It does not in [11,16,28]. The different scaling of temperature is attributed to the effect of gravity acting on density [11,18]. The effect of photofragment excitation produces intermittency in temperature [9]; that intermittency is correlated with temperature itself, Figure 4b. There is a misleading consistency between forecast and climate, macroweather [29], models and the satellite retrievals that feed them. Both are based on the assumption of thermal equilibrium, in the model and in the laboratory spectra upon which the retrievals are based [13]. Temperature is the integrator in the Langevin equation [11] and acts as the middle way between the microscopic and macroscopic [21].

Two centuries of effort have failed to produce turbulence from an analytical, top–down solution to the Navier–Stokes equation [20]. The existing theories have difficulties with the closure—that the *n*th moment depends upon the (*n*+1)th—and with dissipation, which is infrared radiation from the entire atmosphere into the 2.7 K sink of space, not a local downscale cascade from large to small wavelengths (large wavenumbers).

### 4.3. The Turbulent General Circulation

Winds are the transport agent for the general circulation of the atmosphere, which historically was developed as a two-dimensional construct, with latitude–height, zonal mean coordinates considering the system as a thermodynamic engine driven by the surface absorption of solar radiation at low latitudes (<35°) and the emission of infrared radiation into space from higher altitudes at higher latitudes. Flow over topography is still molecules in motion. Angular momentum is not conserved in air with longitudinal gradients, such as in winds, temperature, pressure, and humidity. A zonal mean framework to discuss the entropy of the general circulation is misleading. A more recent treatment used potential vorticity and potential temperature as coordinates to describe the general circulation [30]. The zonal mean basis and local arguments led to acknowledged difficulties, for example, as to why the isentropes were more tightly spaced after cyclogenesis—‘ordered’—than they were before. Potential vorticity is calculated from components which are scale-invariant, and which are subject to radiative decay [31]. The difficulty is a problem arising from not treating the thermodynamic system as consisting of the whole atmosphere, driven by a beam of low entropy solar photons from a blackbody at 5800 K as the organizing energy, with high-entropy infrared radiation over the whole 4π solid angle to the cold sink of space as the dissipation. Since the wind field is turbulent, turbulence drives the general circulation, which pathologically operates in three Cartesian dimensions and 23/9 statistical multifractal dimensions [8,11]. Eady and Sawyer [32,33] would be pleased, albeit down to scales they could not have considered possible. They pointed out that jet streams, circulation ‘cells’ such as the Hadley, Ferrel and Walker variants, should be regarded as secondary phenomena (see Section 9 of [11]).

### 4.4. The Acquisition of Velocity by Winds

The extreme version of the mechanism is the formation of shock waves by lightning strokes. The energy source is the charge separation arising from collisions between ice particles and supercooled water droplets. Cumulonimbus thunderstorms are the phenomenon in which the lightning is embedded; their energy source is warm, moist air at the surface over the land and sea. The organization of the storm is caused by the Gibbs free energy derived from the evaporation of surface water to vapor. Some such storms are sufficiently energetic to act as sources for gamma rays [34,35]. The formation of shock waves occurs when the fastest moving air molecules catch up with each other, forming a front moving at the speed of sound. Dissipation occurs by interaction with the ambient air through which the shock propagates. Tropospheric jet streams are fed by moist air at their equatorward entrance, in which the fastest moving molecules carrying the Gibbs free energy will tend to bunch together, leading to acceleration in the flow. That acceleration will be limited by the tendency of the more numerous average molecules to define an operational temperature as they fill in the volume of lower density air left behind by the fastest ones. Dissipation is effectuated by infrared radiation to space at the operational temperature. A further point is that the rotational energy of all air molecules is likely to be above that of the equilibrated ground state, a feature having a role in the generation of turbulence [20] and ensuring the non-existence of laminar flow at any scale. The higher-speed molecules carrying the Gibbs free energy performing the organizing work will be at a higher number density and therefore at a higher pressure than the more average molecules ahead of them. That will lead to consistency with macroscopic scales in which the pressure gradient causes acceleration.

**Figure 8:**
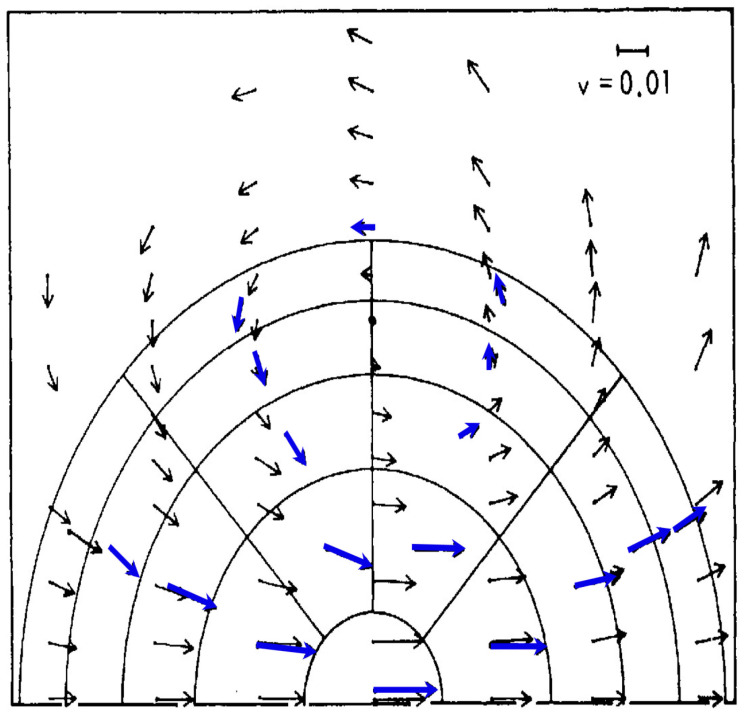
As originally simulated by [2] showing the emergence of hydrodynamic behavior in the form of ‘ring currents’ or vortices in a population of 220 Maxwellian hard spheres impinged upon by an energetic flux of such particles. The blue arrows are the averages of the atom vectors after 9.9 collisions; the black arrows are from a Navier–Stokes simulation. Later simulations showed disagreements between the two approaches. This appears with permission as a high-definition black-and-white Figure 3.1 in [6] and as Figure 1 in [18].

The mechanism is illustrated in Figure 8, involving the persistence of molecular velocity after collision [19] that breaks the continuous translational symmetry of a thermalized air mass. The upscale propagation occurs through the nonlinearity of the mechanism [11,20]. In real air, the leading fast molecules will encounter volumes also possessing molecules with unequilibrated populations—thermalization does not occur as fast as the molecules bunch. In the lower stratosphere and the non-convective troposphere, the energy is provided by hot ozone photofragments [12]. Note that there is considerably more ozone in the troposphere than there was in the late 19th Century [36]. In the upper stratosphere where collisional quenching of excited photofragments is less efficient at the lower pressures, the effects are manifested as a temperature deficit in modeled and analyzed temperatures [12,37]. The atmosphere is of course not homogeneous. For example, it has long been known that the increases in carbon dioxide increase the ozone abundance in the stratosphere [38] via its radiative effects on the temperature dependence of the chemical reactions [39,40].

The application of appropriate, molecular dynamical modeling to the velocities reached in hurricanes and tornadoes, which are difficult to measure but which are thought to be at least 80 ms^−1^ and 130 ms^−1^, respectively [41,42], should constitute an interesting exercise.

**Figure 9:**
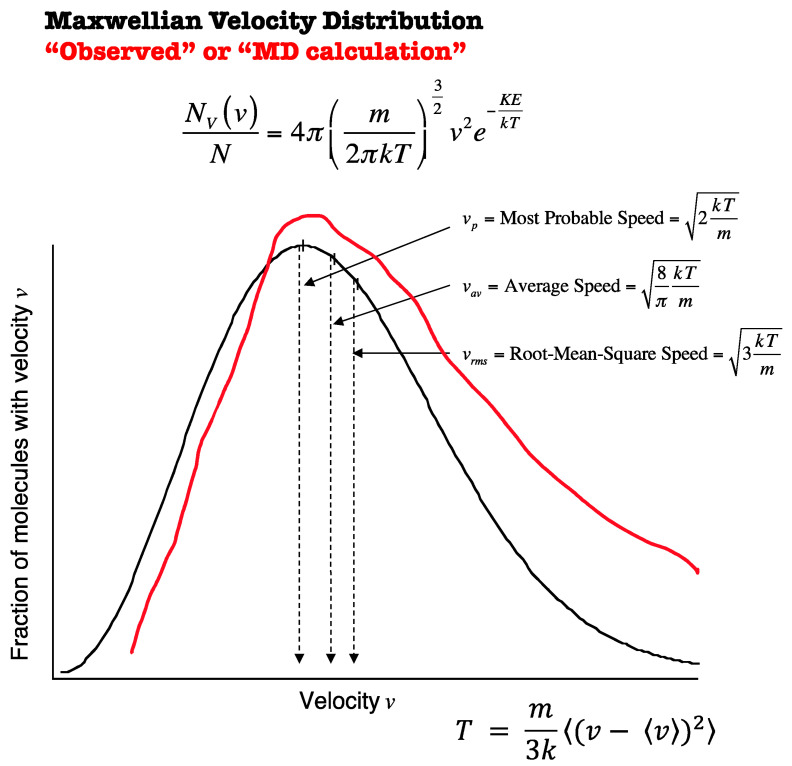
Molecular velocity and temperature. The red curve is hypothetical; it is, however, necessitated by the non-equilibrium status of the real atmosphere as characterized by the content of Figure 3, Figure 4, Figure 5, Figure 6 and Figure 7 above. This appears as Figure 2 in [12].

The energy source driving atmospheric winds is encapsulated in Figure 9. It is the upscale propagation of the energy carried by the most energetic (fastest moving) molecules. The red curve is necessarily hypothetical in the absence of observations [20]. Note that the whole curve, including the low energy values, is shifted towards high values. That is because the real atmosphere has more energy than a hypothetical one at local equilibrium [13]. Line broadening theory is a very complicated quantum mechanical problem. However, using simple impact theory, the greatest displacement from the central energy of the transition between quantum levels will result from the faster molecules. In the atmosphere, the line centers of many water vapor and carbon dioxide lines are self-absorbed, so the far wings are relatively more effective. That enhances the so-called greenhouse effect.

## 5. Conclusions

Winds are molecules moving in a nonrandom, organized way. The large fraction of the energy received by the air goes into maintaining and defining an operational temperature, which is warmer than the equilibrium temperature obtained using Boltzmann statistics [12,13]. The Gibbs free energy defined by scale invariance [14] is the small remaining fraction but is nevertheless effective in providing the organization evident in observations. The dynamical behavior of air molecules translates nonlinearly upscale, with the fastest molecules carrying the Gibbs free energy that drives the wind fields and hence the organized general circulation of the atmosphere. Dissipation, entropy production, is transported by infrared radiation to the cold sink of space via the operational, nonequilibrium temperature of the air. The operation of and conformity with the 2nd Law of Thermodynamics by the entire atmosphere is procured by the turbulent winds driving the general circulation.

## Figures and Tables

**Table 1:** Equivalence between statistical thermodynamic and scaling variables.

Variable	Statistical Thermodynamics	Scaling Equivalent
Temperature	*T*	1/*qk*_Boltzmann_
Partition function	*f*	e^−*K*(*q*)^
Energy	*E*	*γ*
Entropy	−*S*(*E*)	*c*(*γ*)
Gibbs free energy	−*G*	*K*(*q*)/*q*

## Data Availability

Data used are available on the NASA Airborne Database https://earthdata.nasa.gov/ (accessed on 5 June 2021).
